# A large-scale evaluation of therapeutic alliance and symptom trajectories of depression and anxiety in blended care therapy

**DOI:** 10.1371/journal.pone.0313112

**Published:** 2024-11-08

**Authors:** Monica S. Wu, Robert E. Wickham, Shih-Yin Chen, Alethea Varra, Connie Chen, Anita Lungu

**Affiliations:** 1 Lyra Health, Burlingame, CA, United States of America; 2 Department of Psychological Sciences, Northern Arizona University, Flagstaff, AZ, United States of America; Union College, UNITED STATES OF AMERICA

## Abstract

This study sought to conduct a large-scale examination (*N* = 14,951) into the associations between therapeutic alliance and anxiety and depression symptom trajectories within a blended care therapy (BCT) program. Clients receiving blended care services completed weekly outcome measures for anxiety and depression and therapeutic alliance ratings every other therapy session. Using a retrospective, pragmatic study design, latent change score (LCS) analysis captured individual differences in initial therapeutic alliance scores and change in alliance. The LCS variables were specified as predictors of a latent growth curve model describing changes in depression and anxiety symptoms over the course of treatment. Therapeutic alliance scores in the BCT program were generally strong (initial item average = 4.10) and improved over time. Higher initial therapeutic alliance scores and greater initial increases in alliance were associated with steeper declines in anxiety and depressive symptoms at the beginning of therapy. Higher therapeutic alliance (both initial scores and initial increases) was also associated with a deeper symptom trajectory over time, indicating lower anxiety and depressive symptoms overall, as well as sustained decreases in symptoms over time. These results highlight the clinical impact of the working relationship between the provider and the client.

## Introduction

Therapeutic alliance is a multifaceted construct that pertains to the therapeutic relationship between the therapist and the client. Alliance is an important construct that cuts across therapeutic approaches, but its focus may vary depending on the type of therapy [1]. In cognitive-behavioral therapy (CBT), strong therapeutic alliances emphasize collaboration and teamwork [2]. Indeed, key aspects of therapeutic alliance in CBT include agreement on the goals of therapy and the therapeutic tasks, as well as the affective bond between the therapist and client [3]. Given the bidirectional relationship and continual collaboration between the therapist and the client, the therapeutic alliance can also change over time as therapy progresses [4]. As such, therapeutic alliance measured at different timepoints may yield different findings [5], and studies have examined the therapeutic alliance at the beginning, middle, and/or end of treatment [6]. Of note, assessing alliance earlier in treatment can demonstrate greater clinical benefits, as it affords the opportunity for earlier intervention and repair of the relationship if needed. Consequently, many studies have examined therapeutic alliance within the first few sessions of CBT [6–9], often examining its association with later clinical outcomes.

Based on meta-analyses, there has been a consistent, positive association between therapeutic alliance and clinical outcomes across various psychotherapies [6, 10, 11]. More specifically, early therapeutic alliance has been associated with positive clinical outcomes in CBT for adults with depression and anxiety [7, 12–14]. Given the heterogeneity of when alliance has been measured, it is important to consider the implications of the specific timing of the assessments. Earlier assessments at the outset of care may reflect the earlier stages of forming the alliance, while later assessments may tap into more robust alliances that have had time to strengthen. Additionally, therapeutic alliance has predicted the rate of change in symptoms, with better working alliance being associated with a greater rate of improvement in symptoms [15, 16]. Changes in working alliance over time have also been observed to predict changes in symptom severity [15, 17], highlighting the importance of evaluating the impact of early therapeutic alliance as well as changes in alliance over time.

Given the rapidly expanding interest in and need for digital mental health solutions, it is imperative to understand how therapeutic alliance impacts clinical outcomes under these unique circumstances. Therapist-guided internet-based CBT trials have observed similar positive associations between therapeutic alliance and clinical outcomes [18, 19]. However, findings have been more mixed regarding the strength of therapeutic alliance when comparing in-person therapy to teletherapy. On some occasions, the therapeutic alliance has been shown to be stronger in teletherapy [20, 21] or at least equivalent to in-person therapy [22]. Conversely, a recent meta-analysis found the therapeutic alliance to be significantly inferior to face-to-face modalities of therapy, even though the clinical outcomes remained comparable across conditions [23].

There are barriers to therapeutic alliance that are unique to digital mental health solutions in which providers operate remotely (e.g., telehealth, guided iCBT). For instance, providers using videoconferencing may need to make more concerted efforts to create an online presence, notice nonverbal cues, and keep the client engaged in session [24, 25]. These actions are even harder to achieve for interventions that are largely internet- or app-based with minimal provider guidance, further decreasing the opportunity for therapeutic alliances to form. As a solution, blended care forms of therapy (BCT) capitalize on the best of both worlds [26] by fostering stronger therapeutic connections through one-on-one sessions [27], which can be done in person or via teletherapy, and augmenting outcomes with personalized digital content [28, 29]. Indeed, therapeutic alliance has been observed to be equally strong when comparing BCT and traditional face-to-face therapy [30–34]. Strong therapeutic alliance has also been associated with positive clinical outcomes in BCT for depression [16, 30, 33, 35]. However, studies examining these effects for anxiety symptoms are lacking, and the impact of changes in therapeutic alliance over time has yet to be examined in BCT.

To fill these gaps in research, this study seeks to conduct a large-scale examination into the associations between therapeutic alliance scores and anxiety and depression symptom trajectories within a BCT program. Specifically, both the early therapeutic alliance scores and the change in alliance will be examined, and their associations with the initial trajectory and subsequent flattening of symptoms will be evaluated. No studies have comprehensively examined all of these associations within the context of BCT, especially at this scale and with anxiety symptoms (in addition to depression). Conducting these nuanced investigations will help improve our understanding of how therapeutic alliance can impact clinical outcomes in BCT, affording opportunities for targeted intervention and more robust symptom reduction.

## Materials and methods

### Study design and procedures

Using a pragmatic, retrospective study design, this study analyzed data that were collected as part of routine quality assurance for the BCT program at a company that provides workforce mental health benefits. Clients are employees (or their dependents) of companies that offer mental health benefits through the aforementioned company. Clients who were interested in care completed an online triage process, which evaluated their presenting concerns, previous treatment history, and care preferences. Based on their responses, clients were presented with the recommended care services. Clients who were eligible for the BCT program were matched with a provider and able to schedule care thereafter.

All therapy sessions were conducted face-to-face with a provider via videoconference, and providers assigned personalized digital activities (video lessons, digital exercises, digital guides) in between sessions, based on the client’s presenting concerns. Clients also completed weekly symptom severity assessments for anxiety and depression for the duration of care. Therapeutic alliance was assessed at every other therapy session. A change in the assessment protocol during the sampling period resulted in two distinct administration schedules for the therapeutic alliance measure, referred to hereafter as Group 1 and Group 2. Clients in Group 1 completed the therapeutic alliance measure after their odd-numbered therapy sessions (sessions 1, 3, 5, etc.), and clients in Group 2 completed the therapeutic alliance measure after their even-numbered therapy sessions (sessions 2, 4, 6, etc.). A systematic change in the timing of the working alliance assessment used in the present study allows examination of how the magnitude and variability of change in therapeutic alliance may differ when measured in sessions 1 and 3, versus 2 and 4.

All therapy sessions, digital activities, and assessments were completed via a secure, Health Insurance Portability and Accountability Act (HIPAA)–compliant platform that is proprietary to the aforementioned company. Given that this is a retrospective analysis of routinely collected and deidentified data, this study was determined to be exempt research by the Western-Copernicus Group (WCG) Institutional Review Board.

### Participants and data inclusion

Participants started BCT treatment between May 15, 2020, and June 16, 2022. To be included in the BCT program, clients must have been 18 years of age or older and willing to participate in therapy sessions over video. Clients were not eligible for the BCT program if they reported active suicidality, homicidality, or self-harm, or if they had a current diagnosis of severe alcohol/substance use disorder(s), unstable bipolar disorder, or psychiatric disorder with psychotic features that were not stabilized by medications. The BCT program has sought and obtained written, informed consent from all participants in the research. This research did not include minors.

Participants who scored above the clinical cutoff for either the Generalized Anxiety Disorder-7 (GAD-7; score ≥8) or the Patient Health Questionnaire-9 (PHQ-9; score ≥10) on a valid baseline assessment (N = 20,452) were eligible for this study. Baseline assessments were considered invalid if they were collected more than 2 weeks prior to the first therapy session or after the second therapy session with the provider. In addition, participants were considered to be missing a valid second assessment if no additional assessment was completed within 5 weeks after the last therapy session. Assessments were also excluded if they were collected more than 15.7 weeks after the first therapy session, which represents the mean plus one standard deviation of the treatment duration for the sample.

**Fig 1:**
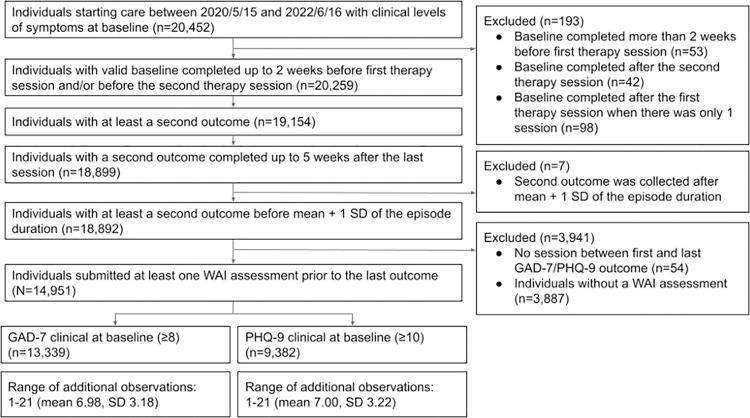
Participant flow. GAD-7: Generalized Anxiety Disorder-7; PHQ-9: Patient Health Questionnaire-9.

Additionally, participants were included only when at least one Working Alliance Inventory—Short Revised (WAI-SR) was collected prior to the completion of the last assessment, resulting in a final sample size of *N* = 14,951. A change in the assessment protocol of WAI during the sampling period produced two distinct groups of clients (Group 1 and Group 2). Episodes for clients in Group 1 began between May 15, 2020 and March 10, 2021 (*n* = 2,966), and episodes for clients in Group 2 began between March 11, 2021 and June 16, 2022 (n = 11,985). A comprehensive diagram of the participant flow is demonstrated in Fig 1.

### Blended care therapy program

The BCT program has been previously described in detail [36], but it can be summarized as combined live video-based sessions with a therapist plus between-session digital care tools (i.e., lessons and exercises, therapist feedback, assessments). Providers conducted therapy sessions via a secure, proprietary HIPAA-compliant video platform developed by the aforementioned company, and all sessions were virtual. Treatment generally started with weekly sessions and gradually titrated to biweekly sessions. Providers assigned digital activities through the platform, and these activities were collaboratively chosen and personalized based on the client’s presenting issues. Providers were also available via asynchronous messaging to answer questions, provide feedback, and clarify content.

Therapists consisted of 687 licensed therapists (licensed clinical psychologists, licensed marriage and family therapists, licensed clinical social workers, or licensed professional counselors). Cognitive behavioral and transdiagnostic approaches were used in therapy, including the Unified Protocol [37], Acceptance and Commitment Therapy [38], and Dialectical Behavior Therapy [39]. Providers received intensive training in CBT and on the proprietary online platform. Ongoing quality assurance was conducted via random session video reviews, regular consultation meetings, and continuing education presentations.

### Digital activities

The digital activities in the BCT program entailed digital video lessons, digital exercises, and/or digital guides. All digital activities were rooted in evidence-based treatments and designed by clinical psychologists who integrated information from multiple resources (e.g., treatment manuals, clinical expertise, research studies) to develop the activities. *Digital video lessons* used a storytelling format to present key CBT-informed concepts and skills, enhancing reliability and normalization of mental health challenges [40]. Digital video lessons followed a character on their care journey, covering a variety of clinical topics such as thinking traps, mindful awareness, and effective communication. Each video lesson was about 10 minutes long, and clients completed a brief quiz at the end of the video to check for understanding. *Digital exercises* were digitized versions of traditional worksheets used in therapy to facilitate out-of-session practice of therapeutic skills. Digital exercises ranged from awareness-building activities (e.g., log to document thoughts, feelings, and behaviors) to practice-oriented activities (e.g., exposure practice, behavioral activation log). *Digital guides* were analogues of traditional handouts provided in therapy that help reinforce key CBT-based concepts and skills and provide more in-depth context for a particular topic. Example digital guide topics include setting boundaries or understanding trauma.

### Measures

#### Demographic information

Demographic information regarding the client’s sex, ethnicity, and age were obtained from the initial intake assessment, which was completed by the client via the online platform prior to the first therapy session.

## Therapeutic alliance

The Working Alliance Inventory—Short Revised (WAI-SR) is a 12-item self-report questionnaire that evaluates the client’s perception of the therapeutic alliance with the provider based on the goals (agreement on goals of therapy), tasks (agreement on the therapeutic tasks), and the bond (affective bond between the therapist and client) [41]. Responses are rated on a Likert scale from 1 (*Seldom*) to 5 (*Always*), with higher scores indicating stronger alliance. A total score is created by summing all responses. The WAI-SR has demonstrated strong reliability and validity across outpatient and inpatient settings within a variety of presenting issues [3, 41]. Cronbach’s alpha [42] was used to examine the reliability of the initial WAI score for the entire sample. The Cronbach’s alpha values of the initial WAI for Group 1 and Group 2 were .94 and .93, respectively.

## Symptom severity assessments

The Generalized Anxiety Disorder-7 (GAD-7) is a 7-item self-report questionnaire that evaluates the presence and severity of anxiety symptoms [43]. A total score of ≥8 on the GAD-7 is indicative of a likely anxiety disorder diagnosis [44]. The Patient Health Questionnaire-9 (PHQ-9) is a 9-item self-report questionnaire that evaluates the presence and severity of depressive symptoms. A score of ≥10 on the PHQ-9 indicates a likely diagnosis of major depression [45].

Both the GAD-7 and PHQ-9 are rated on a scale from 0 to 3, with higher scores reflecting more severe symptoms. These measures have been evaluated extensively across numerous studies, exhibiting strong reliability and validity [46], as well as good treatment sensitivity [47]. Cronbach’s alpha was calculated to report the reliability of both GAD-7 and PHQ-9 score at baseline for the entire sample. The reliability coefficients of the baseline GAD-7 and the baseline PHQ-9 were .74 and .77, respectively.

### Data analysis strategy

**Fig 2:**
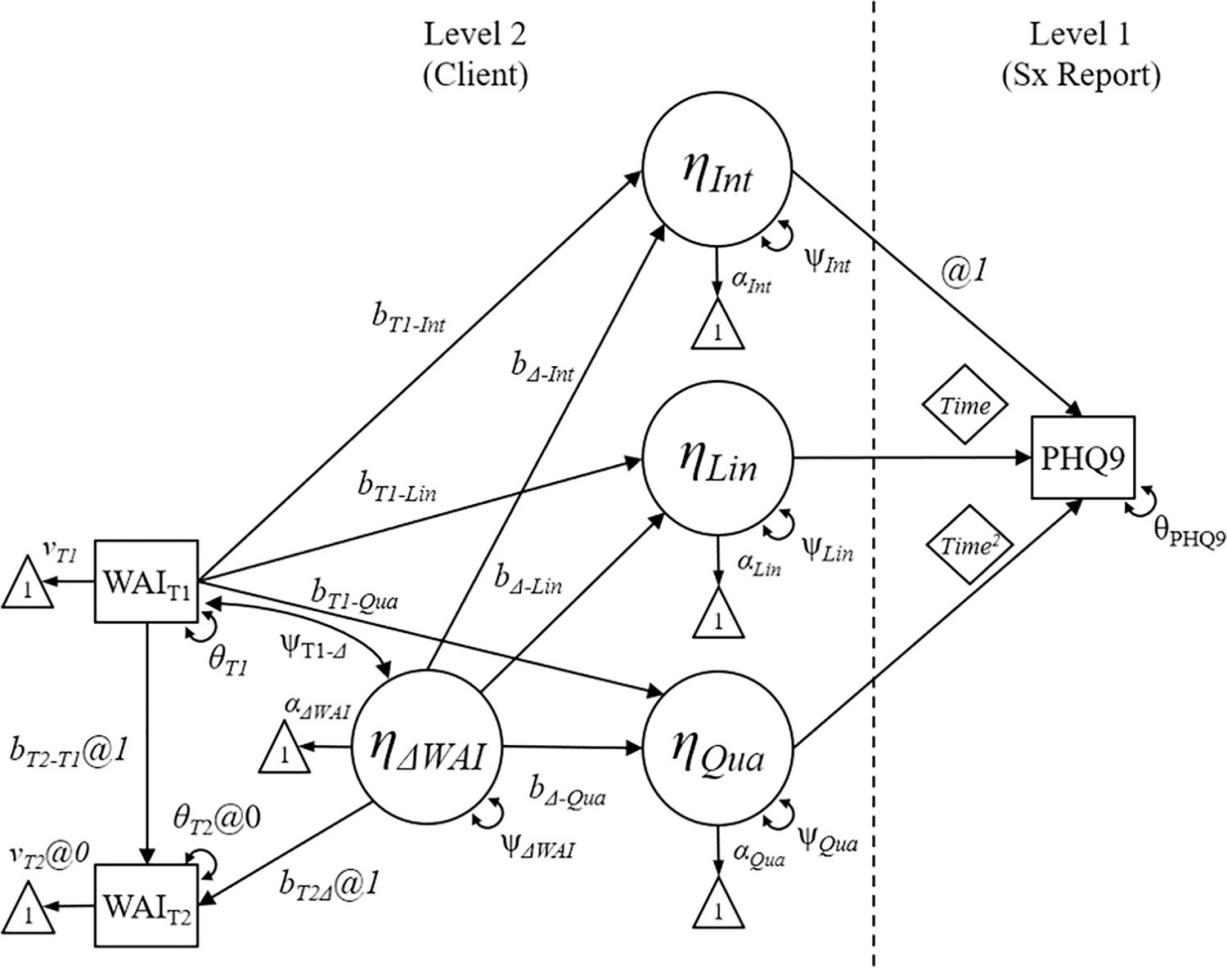
Structural equation model path diagram.

Multiple-Group Multilevel Structural Equation Modeling [48] was used to examine the association between initial WAI and change in WAI scores across clients (Level 2) and individual differences in clients’ depression and anxiety symptom trajectories (Level 1) over the course of a treatment episode. Differences in WAI change scores were modeled using a Latent Change Score (LCS) approach [49, 50], which is illustrated on the left side of Fig 2. The LCS model uses repeated assessments (WAI_T1_, WAI_T2_) from the same person to estimate individual change scores in the form of a latent variable (η_ΔWAI_) representing the estimated difference in WAI across the two measurement occasions. The focal parameters in the LCS are the means (ν_WAI-T1_, α_ΔWAI_) describing the average score at the first time point (WAI_T1_) and the average change score, as well as the variances (θ_WAI-T1_, ψ_ΔWAI_) of WAI_T1_ and the latent change variable η_ΔWAI_. Although it is possible to calculate observed change scores (i.e., WAI_T2-_WAI_T1_), this approach is problematic because it would result in a missing value for cases with only one WAI score present. In contrast, the LCS approach leverages missing data handling capabilities of the full-information ML estimator used in Mplus 8.8 [51].

The latent change variable (η_ΔWAI_), and the initial measurement occasion (WAI_T1_) may be used to predict other variables representing attributes of the client. The middle of Fig 2 contains latent variables representing the client-level random effects associated with a quadratic latent curve model describing individual differences in initial depression (PHQ-9) or anxiety (GAD-7) symptoms (η_Int_), the initial rate of change in symptoms (η_Lin_), and the shape of the symptom trajectory (η_Qua_). A quadratic term was integrated into the growth curve model based on previously published research that examined similar symptom trajectories [29, 36, 52]; these studies have consistently found significant quadratic components, reflecting the necessity of including a quadratic term for accurate model specification. The regression paths linking the LCS variables (WAI_T1_, η_ΔWAI_) to the latent curve model components describe the degree to which clients who report higher initial WAI or more positive initial change in WAI, report higher or lower levels of initial PHQ-9 or GAD-7 (b_T1-Int_, b_Δ-Int_), more positive or negative initial change in symptoms (b_T1-Lin_, b_Δ-Lin_), and a deeper or shallower symptom trajectory (b_T1-Qua_, b_Δ-Qua_). Finally, the right side of Fig 2 consists of the observed symptom report (PHQ-9, GAD-7) on a given response occasion (*i*) for a client (*j*).

Differences in the timing of WAI assessments across protocols (i.e., Group 1 vs. Group 2) were accommodated using a multiple-group model. To improve the interpretability of the growth curve parameters describing the average trajectory (i.e., α_Int_, α_Lin_, α_Qua_), WAI_T1_ and WAI_T2_ scores were centered around the WAI_T1_ mean separately for each Group. Although this approach discards across-Group differences in WAI_T1_, it preserves differences in the magnitude of initial WAI change and produces conditional growth parameters that correspond to a client with an average WAI_T1_ score and no change in WAI (i.e., η_ΔWAI_ = 0). The mean (ν_WAI-T1_, α_ΔWAI_) and variance (θ_WAI-T1_, ψ_ΔWAI_) parameters for the LCS component of the model were expected to differ across Group 1 and Group 2 groups, and the possibility that the paths linking the LCS variables to the latent curve variables (b_T1-Int_, b_Δ-Int_, b_T1-Lin_, b_Δ-Lin_, b_T1-Qua_, b_Δ-Qua_) may also differ across groups was examined. This was accomplished by specifying an unconstrained multiple-group model and creating auxiliary parameters representing the difference between the Group 1 and Group 2 estimates for each of the above parameters.

In addition to the nesting of response occasions (Level 1) within clients (Level 2), clients were also nested within treatment providers (Level 3). The paucity of variance at the provider level creates challenges when attempting to specify a 3-level model, so the small degree of clustering across providers was accommodated using the COMPLEX option, which uses a sandwich estimator to adjust the standard errors [53]. More specifically, the COMPLEX option accounts for the non-independence in WAI and symptom reports that may arise from the nesting of clients within providers, ensuring that the standard errors (and associated significance tests) are unbiased.

## Results

### Descriptive analysis

The baseline characteristics and treatment duration of the entire sample, as well as of the clinical subgroups (GAD-7 sample and PHQ-9 sample), can be found in Table 1. Episode duration refers to the length of the episode of care in weeks, starting from the initial intake session and ending with the final treatment session that occurred before the last outcome assessment (after outlier truncation). The average score for the initial WAI administration was 4.10. P-values were calculated with t-tests for numerical variables and with chi-squared tests for categorical variables. Number of therapy sessions was compared using Poisson regressions.

**Table 1:** Baseline characteristics and treatment duration.

			GAD-7 Sample				PHQ-9 Sample			
		Entire Sample	Group 1	Group 2	p-value (t or χ2)	Effect Size	Group 1	Group 2	p-value (t or χ2)	Effect Size
n		14951	2656	10683			1748	7634		
Age, mean (SD)		33.41 (9.25)	33.21 (8.90)	33.40 (9.29)	0.332		33.34 (9.01)	33.38 (9.44)	0.861	-0.005
Gender, n (%)					0.049	0.021			0.062	0.024
	Female	9829 (65.74)	1741 (65.55)	7055 (66.04)			1123 (64.24)	4981 (65.25)		
	Male	4973 (33.26)	899 (33.85)	3510 (32.86)			614 (35.13)	2561 (33.55)		
	Other/unknown	149 (1.00)	16 (0.60)	118 (1.10)			11 (0.63)	92 (1.21)		
Race/ethnicity, n (%)					<0.001	0.089			<0.001	0.090
	White	7441 (49.77)	1204 (45.33)	5436 (50.88)			758 (43.36)	3742 (49.02)		
	Asian or Pacific Islander	3010 (20.13)	694 (26.13)	1982 (18.55)			449 (25.69)	1431 (18.75)		
	Hispanic or Latino	1476 (9.87)	254 (9.56)	1067 (9.99)			174 (9.95)	834 (10.92)		
	Multiple	1262 (8.44)	220 (8.28)	914 (8.56)			160 (9.15)	675 (8.84)		
	Black or African American	1020 (6.82)	122 (4.59)	774 (7.25)			92 (5.26)	613 (8.03)		
	Other	231 (1.55)	51 (1.92)	155 (1.45)			34 (1.95)	105 (1.38)		
	American Indian or Alaska Native	23 (0.15)	4 (0.15)	16 (0.15)			3 (0.17)	13 (0.17)		
	Native Hawaiian or Other Pacific Islander	17 (0.11)	3 (0.11)	10 (0.09)			1 (0.06)	12 (0.16)		
	Missing or Prefer not to disclose	471 (3.15)	104 (3.92)	329 (3.08)			77 (4.41)	209 (2.74)		
Highest education, n (%)					<0.001	0.116			<0.001	0.116
	College graduate or above	10674 (71.39)	2133 (80.31)	7396 (69.23)			1342 (76.77)	4960 (64.97)		
	No college degree	3156 (21.11)	463 (17.43)	2349 (21.99)			366 (20.94)	1989 (26.05)		
	Missing	1121 (7.50)	60 (2.26)	938 (8.78)			40 (2.29)	685 (8.97)		
Employment status, n (%)					0.021	0.024			0.006	0.033
	Employed	13848 (92.62)	2440 (91.87)	9904 (92.71)			1583 (90.56)	7061 (92.49)		
	Unemployed	1065 (7.12)	214 (8.06)	747 (6.99)			163 (9.32)	552 (7.23)		
	Missing	38 (0.25)	2 (0.08)	32 (0.30)			2 (0.11)	21 (0.28)		
Financial status, n (%)					<0.001	0.071			<0.001	0.081
	Good	8445 (56.48)	1678 (63.18)	5867 (54.92)			1037 (59.32)	3752 (49.15)		
	Fair	5362 (35.86)	833 (31.36)	3927 (36.76)			590 (33.75)	3123 (40.91)		
	Poor	819 (5.48)	112 (4.22)	627 (5.87)			95 (5.43)	595 (7.79)		
	Missing	325 (2.17)	33 (1.24)	262 (2.45)			26 (1.49)	164 (2.15)		
Baseline GAD-7, mean (SD)		12.04 (4.18)	12.57 (3.50)	12.92 (3.64)	<0.001	-0.097	12.11 (4.63)	12.39 (4.72)	0.023	-0.060
Baseline PHQ-9, mean (SD)		11.26 (5.12)	10.49 (5.10)	11.20 (5.33)	<0.001	-0.134	13.97 (3.48)	14.42 (3.68)	<0.001	-0.121
Initial WAI, mean (SD)		49.23 (8.30)	46.95 (9.25)	49.90 (7.92)	<0.001	-0.360	46.74 (9.47)	49.73 (7.99)	<0.001	-0.361
Number of therapy sessions, mean (SD)		6.39 (2.52)	6.43 (2.74)	6.39 (2.46)	0.432		6.57 (2.76)	6.42 (2.52)	0.027	-0.059
Episode duration (weeks), mean (SD)		7.86 (3.83)	7.83 (4.06)	7.86 (3.77)	0.723		7.99 (4.09)	7.83 (3.80)	0.146	

### Primary analysis

The conditional latent growth curve model illustrated in Fig 2 was estimated for the GAD-7 and PHQ-9 symptom reports. The final models converged without error.

### GAD-7

Model parameters describing the GAD-7 analysis are provided in Table 2. Clients in both Group 1 (α_ΔWAI-Group1_ = 4.320 [3.984, 4.656]) and Group 2 (α_ΔWAI-Group2_ = 2.774 [2.640, 2.907]) exhibited a notable increase in WAI scores, though the magnitude of average increase was significantly larger for Group 1 (Est_Diff_ = -1.546 [-1.908, -1.185],). In addition, clients in both Groups exhibited comparable levels of baseline anxiety symptoms (α_Int-Group1_ = 10.913 [10.761, 11.065]; α_Int-Group2_ = 11.239 [11.155, 11.322]), and clients in both Groups showed an initial decline in symptoms of approximately 1 unit per week (α_Lin-Group1_ = -1.093 [-1.149, -1.037]; α_Lin-Group2_ = -1.131 [-1.160, -1.102]), and the quadratic component indicated that this decline became weaker (more positive) over the course of treatment (α_Qua-Group1_ = 0.054 [0.049, 0.058]; α_Qua-Group2_ = 0.055 [0.053, 0.057]). The regressions linking WAI_T1_ (b_T1-Int-Group1_ = 0.011 [-0.005, 0.028]; b_T1-Int-Group2_ = 0.002 [-0.008, 0.012]) and η_ΔWAI_ (b_Δ-Int-Group1_ = 0.015 [-0.009, 0.039]; b_Δ-Int-Group2_ = 0.008 [-0.008, 0.024]) to the intercept component failed to reach significance in both Group 1 and Group 2. However, the coefficients connecting the LCS variables to the linear growth component suggested that clients in both Groups who reported higher initial WAI_T1_ (b_T1-Lin-Group1_ = -0.020 [-0.026, -0.014]; b_T1-Lin-Group2_ = -0.024 [-0.027, -0.021]) and more positive change in WAI (b_Δ-Lin-Group1_ = -0.019 [-0.027, -0.011]; b_Δ-Lin-Group2_ = -0.026 [-0.031, -0.021]) exhibited a steeper (more negative) initial trajectory. In addition, the paths between the LCS variables and the quadratic growth component indicated that clients in both Groups who reported higher initial WAI_T1_ (b_T1-Qua-Group1_ = 0.001 [0.001, 0.002]; b_T1-Qua-Group2_ = 0.001 [0.001, 0.002]) and more positive change in WAI (b_Δ-Qua-Group1_ = 0.001 [0.001, 0.002]; b_Δ-Qua-Group2_ = 0.002 [0.001, 0.002]) had deeper symptom trajectories (i.e., a lower minimum). All Wald Z tests comparing the magnitude of these coefficients across Group 1 and Group 2 failed to reach significance (all ps > .13).

**Table 2:** Key parameters from GAD-7 analysis.

	Group 1			Group 2		
Parm	Est [95% CI]	Z	p-value	Est [95% CI]	Z	p-value
*α*_*ΔWAI*_	4.320 [3.984, 4.656]	25.203	<0.001	2.774 [2.640, 2.907]	40.642	<0.001
*α*_*Int*_	10.913 [10.761, 11.065]	140.756	<0.001	11.239 [11.155, 11.322]	264.647	<0.001
*α*_*Lin*_	–1.093 [–1.149, –1.037]	–38.321	<0.001	–1.131 [–1.160, –1.102]	–76.321	<0.001
*α*_*Qua*_	0.054 [0.049, 0.058]	24.479	<0.001	0.055 [0.053, 0.057]	47.067	<0.001
*b*_*T1-Int*_	0.011 [–0.005, 0.028]	1.353	0.176	0.002 [–0.008, 0.012]	0.429	0.668
*b*_*Δ-Int*_	0.015 [–0.009, 0.039]	1.237	0.216	0.008 [–0.008, 0.024]	0.949	0.342
*b*_*T1-Lin*_	–0.020 [–0.026, –0.014]	–6.745	<0.001	–0.024 [–0.027, –0.021]	–15.151	<0.001
*b*_*Δ-Lin*_	–0.019 [–0.027, –0.011]	–4.864	<0.001	–0.026 [–0.031, –0.021]	–10.536	<0.001
*b*_*T1-Qua*_	0.001 [0.001, 0.002]	5.622	<0.001	0.001 [0.001, 0.002]	10.820	<0.001
*b*_*Δ-Qua*_	0.001 [0.001, 0.002]	4.096	<0.001	0.002 [0.001, 0.002]	7.712	<0.001

In sum, clients in the elevated anxiety sample generally experienced increases in therapeutic alliance over time, with clients in Group 1 demonstrating a larger average increase in alliance. Clients demonstrated significant decreases in anxiety symptoms over the course of treatment, with steeper decreases early on and continued decreases over time at a slower rate. Initial therapeutic alliance and changes in therapeutic alliance were not related to baseline anxiety symptom severity. Clients who had higher initial therapeutic alliance and more positive changes in alliance demonstrated steeper decreases in anxiety symptoms early on in treatment. Similarly, higher therapeutic alliance (both initial scores and increases) was also associated with a steeper symptom trajectory over time, indicating lower anxiety symptoms overall, as well as sustained decreases in anxiety over time.

### PHQ-9

A similar pattern of findings (Table 3) for the latent change score parameters emerged in the PHQ-9 sample for Group 1 (α_ΔWAI-Group1_ = 4.418 [4.003, 4.833]) and Group 2 (α_ΔWAI-Group2_ = 2.838 [2.669, 3.008]), and the average change estimates were also significantly stronger in Group 1 (Est_Diff_ = -1.579 [-2.028, -1.131]). Clients in both Groups exhibited comparable levels of baseline depression symptoms (α_Int-Group1_ = 11.990 [11.770, 12.210]; α_Int-Group2_ = 12.430 [12.326, 12.534]). In addition, clients in both Groups showed an initial decline in depression of approximately 1 unit per week (α_Lin-Group1_ = -1.256 [-1.327, -1.186]; α_Lin-Group2_ = -1.325 [-1.361, -1.288]), and the quadratic component indicated that this decline became weaker (more positive) over the course of treatment (α_Qua-Group1_ = 0.062 [0.056, 0.067]; α_Qua-Group2_ = 0.065 [0.062, 0.068]). The coefficients linking WAI_T1_ (b_T1-Int-Group1_ = -0.016 [-0.039, 0.008]; b_T1-Int-Group2_ = -0.005 [-0.018, 0.009]) and η_ΔWAI_ (b_Δ-Int-Group1_ = 0.018 [-0.012, 0.049]; b_Δ-Int-Group2_ = 0.017 [-0.003, 0.036]) to the intercept component failed to reach significance in both Groups. As before, the coefficients connecting the LCS variables to the linear growth component suggested that clients in both Groups reporting higher WAI_T1_ (b_T1-Lin-Group1_ = -0.017 [-0.024, -0.009]; b_T1-Lin-Group2_ = -0.029 [-0.033, -0.024]) and more positive change (b_Δ-Lin-Group1_ = -0.024 [-0.034, -0.014]; b_Δ-Lin-Group2_ = -0.029 [-0.035, -0.022]) showed a steeper (more negative) initial trajectory. Finally, the paths between the LCS variables and the quadratic growth component indicated that clients in both Groups who reported higher initial WAI_T1_ (b_T1-Qua-Group1_ = 0.001 [0.000, 0.001]; b_T1-Qua-Group2_ = 0.002 [0.001, 0.002]) and more positive change (b_Δ-Qua-Group1_ = 0.001 [0.001, 0.002]; b_Δ-Qua-Group2_ = 0.002 [0.001, 0.002]) had deeper symptom trajectories. Two of the Wald tests comparing the strength of these relationships across groups revealed significant differences. Specifically, the b_T1-Lin_ (Est_Diff_ = -.012 [-.021, -.004]) and b_T1-Qua_ (Est_Diff_ = .001 [.000, .001]) coefficients were stronger in Group 2, relative to Group 1.

**Table 3:** Key parameters from PHQ-9 analysis.

	Group 1			Group 2		
Parm	Est [95% CI]	Z	p-value	Est [95% CI]	Z	p-value
*α*_*ΔWAI*_	4.418 [4.003, 4.833]	20.875	<0.001	2.838 [2.669, 3.008]	32.755	<0.001
*α*_*Int*_	11.990 [11.770, 12.210]	106.982	<0.001	12.430 [12.326, 12.534]	234.442	<0.001
*α*_*Lin*_	–1.256 [–1.327, –1.186]	–34.715	<0.001	–1.325 [–1.361, –1.288]	–71.237	<0.001
*α*_*Qua*_	0.062 [0.056, 0.067]	21.888	<0.001	0.065 [0.062, 0.068]	44.304	<0.001
*b*_*T1-Int*_	-0.016 [–0.039, 0.008]	–1.293	0.196	–0.005 [–0.018, 0.009]	–0.663	0.507
*b*_*Δ-Int*_	0.018 [–0.012, 0.049]	1.185	0.236	0.017 [–0.003, 0.036]	1.653	0.098
*b*_*T1-Lin*_	–0.017 [–0.024, –0.009]	–4.373	<0.001	–0.029 [–0.033, –0.024]	–12.594	<0.001
*b*_*Δ-Lin*_	–0.024 [–0.034, –0.014]	–4.600	<0.001	–0.029 [–0.035, –0.022]	–9.146	<0.001
*b*_*T1-Qua*_	0.001 [0.000, 0.001]	3.437	<0.001	0.002 [0.001, 0.002]	10.005	<0.001
*b*_*Δ-Qua*_	0.001 [0.001, 0.002]	3.771	<0.001	0.002 [0.001, 0.002]	7.016	<0.001

In sum, clients in the elevated depressive symptoms sample generally experienced increases in therapeutic alliance over time, with clients in Group 1 demonstrating a larger average increase in alliance. Clients demonstrated significant decreases in depressive symptoms over the course of treatment, with steeper decreases early on and continued decreases over time at a slower rate. Initial therapeutic alliance and changes in therapeutic alliance were not related to baseline depressive symptom severity. Clients who had higher initial therapeutic alliance and more positive changes in alliance demonstrated steeper decreases in depressive symptoms early on in treatment. Similarly, higher therapeutic alliance (both initial scores and increases) was also associated with a steeper symptom trajectory over time, indicating lower depressive symptoms overall, as well as sustained decreases in depressive symptoms over time.

### Illustration of covariate effects

**Fig 3:**
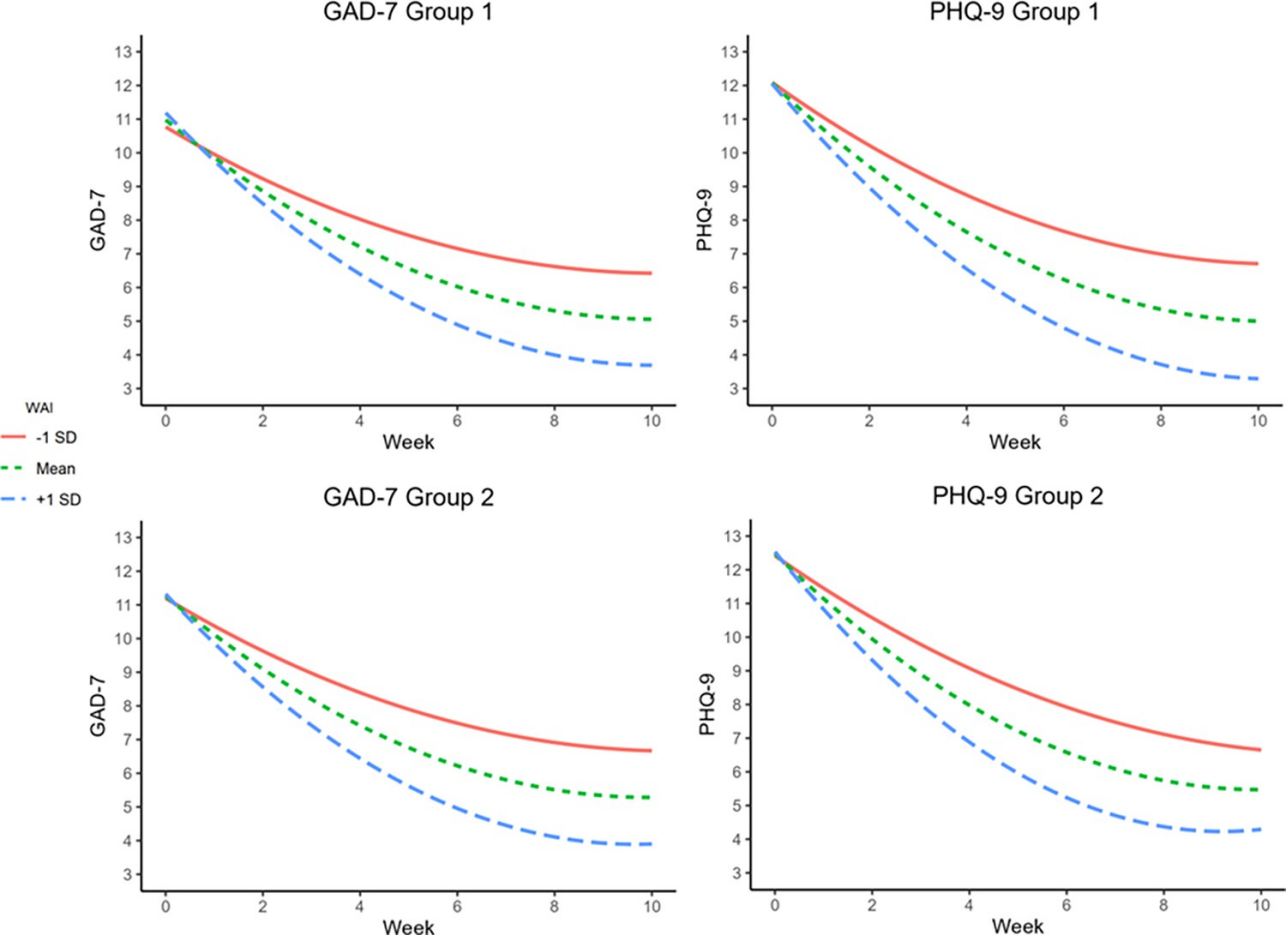
Initial therapeutic alliance and symptom trajectories across groups and symptoms. WAI values describe the conditional moderating effect of both WAI_T1_ and η_ΔWAI_ at the value listed.

The impact of initial WAI and change in WAI on predicted symptom trajectories was evaluated by calculating conditional trajectories at benchmark values of the covariates [54]. Specifically, conditional regression equations were calculated at ± 1 SD around the mean WAI_T1_ and η_ΔWAI_ values for each Group, and the ggplot2 library [55] in R 4.2.1 [56] was used to generate the conditional trajectories illustrated in Fig 3. Trajectory plots for the GAD-7 analysis are provided in the upper (Group 1) and lower (Group 2) left panels, and the PHQ-9 analysis plots are located on the right. The impact of the covariate effects linking WAI_T1_ and η_ΔWAI_ to the growth parameters is evident in the divergence of the trajectories for the low (-1 SD), average (mean), and high (+1 SD), with the trajectory associated with low WAI_T1_ and η_ΔWAI_ benchmark values following a shallower (less concave) path, relative to the trajectories for the mean and high WAI_T1_ and η_ΔWAI_ benchmark values.

## Discussion

The therapeutic alliance in the context of a BCT program was strong, demonstrating an initial average score that was higher than those reported by several studies evaluating blended care programs [16, 31], internet-based CBT [57], and face-to-face individual therapy [58]. Additionally, therapeutic alliance was observed to notably improve over time for clients with elevated anxiety and/or depressive symptoms. Group 1 clients experienced a greater average improvement in therapeutic alliance overall, which is expected given that their alliance scores were taken 1 session earlier than Group 2 clients, thus allowing for increased opportunities to spend time with the provider and form stronger alliances. Overall, these findings support the advantageous synergy of blending face-to-face sessions with digital clinical activities in the context of therapeutic alliance. To achieve the robust provider-client relationships observed in this program, it was likely important to root the digital activities in evidence-based practices, create engaging digital content, and achieve high quality care through thoughtful training and ongoing supervision.

Baseline symptom severity was not associated with initial therapeutic alliance nor change in alliance, which is consistent with previous findings that baseline symptom severity did not predict therapeutic alliance later in care [59]. Alternatively, strong therapeutic alliance scores at the beginning of care were associated with better outcomes in therapy, which is supported by the extant literature [16, 30, 33, 35]. Specifically, greater therapeutic alliance and more positive changes were linked to steeper decreases in anxiety and depressive symptoms at the beginning of care. Indeed, if the provider and client are able to form a strong relationship and instill hope early on, clients are likely to be more motivated to buy into the therapeutic process and experience quicker decreases in their symptom severity.

Greater therapeutic alliance (both initial scores and improvements over time) was associated with lower anxiety and depressive symptoms overall, as well as sustained decreases in symptomatology over time. These findings may in large part be attributed to stronger therapeutic alliances being associated with better mutual alignment on therapy goals and clinical skills [60]. This often translates to greater client commitment to practice therapeutic tasks and assignments [61], which is a key component to generalizing skills and maintaining treatment gains. Interestingly, Group 2 clients with elevated depressive symptoms at baseline observed steeper initial decreases in symptomatology and deeper symptom trajectories compared to Group 1 clients. Clients in Group 2 had their therapeutic alliance assessed one session later than those in Group 1, which could be viewed as a more representative evaluation of the alliance, ultimately serving as a more robust predictor of the clinical outcomes. The sample size differences may have also strengthened the ability to reliably obtain estimates for the complex interaction effects in the Group 2 clients.

The findings of this study should be noted within several limitations and considerations for future directions. First, this study used a pragmatic retrospective design, which precludes our ability to definitively establish inferences of causality and directionality in our findings. Although we used SEM and multiple-timepoint assessments to strengthen our analyses, future studies are recommended to employ a randomized controlled trial to maximize the robustness of the study design (e.g., comparing BCT to traditional face-to-face therapy). Second, this study focused on the therapeutic alliance scores towards the beginning of treatment, given that clients ranged in their length of care and choosing early timepoints facilitated the most inclusive sample. Of note, the results suggest that initial therapeutic alliance and change in scores are predictive of symptom trajectories, regardless of when alliance was measured. Nevertheless, future studies should consider investigating additional assessments of therapeutic alliance, especially later in care, to determine whether there are differences in findings based on the timing of therapeutic alliance measures. Third, this study only employed client-reported therapeutic alliance scores. Future studies should seek to evaluate therapist- and observer-reported therapeutic alliance to determine whether there are differences in findings based on respondent [1]. Fourth, future work remains on how to optimize therapeutic alliance when there are early signals of misalignment or conflict. Further work is needed to evaluate the efficacy of targeted interventions to help strengthen the alliance, especially towards the beginning of care. Fifth, disentangling the contributions of the digital components of technology-enhanced care can also help improve our understanding of therapeutic alliance within this context. Those findings could help inform certain technological advancements or features to help strengthen alliance (e.g., platform messaging function to connect the provider and client). While the present study is able to examine the extent of messaging between the provider and client on a descriptive level (*M* = 3.30, *SD* = 2.64 messages between the provider and client per session), it was not possible to parse out how much of the messaging was dedicated to expected logistics (e.g., rescheduling, confirming appointments) and how much of it was dedicated to therapeutic support (e.g., problem-solving an issue with skills practice). As such, future studies could benefit from examining this in more detail and crystallizing the unique role asynchronous messaging may play in the therapeutic relationship. Sixth, the present study sample overlapped with the emergence and stabilization of the COVID pandemic. There is not a clear operationalization of when COVID timing “started” and “stabilized,” and the timing of it is co-confounded by the change in WAI administration schedule. Although COVID timing is not believed to be theoretically relevant to the goals of the study and the conclusions drawn from the model results, and the timing of our change in WAI administration schedule also accommodates (and may also capture) an impact of COVID, it is noted that our present model does not account for COVID timing.

## Conclusions

As a notable strength to this study, a widely validated measure of therapeutic alliance was utilized with an unprecedented sample size, and found that the therapeutic alliance was robust and improved over time. Additionally, this is the first study to examine these complex associations between initial therapeutic alliance and anxiety/depression symptom trajectories in BCT. These nuanced investigations into therapeutic alliance are important, as CBT-based treatments encourage the client to break out of maladaptive cycles and embrace challenging situations; having buy-in and alignment early on in care will optimize client engagement and motivation to persist with difficult therapeutic tasks. Additionally, a strong provider-client bond will allow for flexibility and troubleshooting whenever challenges emerge in care, facilitating perseverance in the face of adversity and sustained treatment gains. If ruptures occur in the alliance or there appears to be difficulty establishing a strong therapeutic connection, extra efforts should be dedicated to strengthening the relationship before moving forward in care. In particular, technology could play an impactful role in providing timely feedback to providers and flagging these instances as they arise, empowering the provider to proactively address these challenges. Ultimately, these findings highlight the importance of cultivating a strong relationship between the provider and the client, given its notable impact on the trajectory and overall severity of symptoms within a blended care context.

## Supporting information

S1 Text Auxiliary analysis incorporating therapy homework completion. (DOCX)

S1 Table Key parameters from GAD-7 analysis. (DOCX)

S2 Table Key parameters from PHQ-9 analysis. (DOCX)
