# Supplementary material for: A large-scale evaluation of therapeutic alliance and symptom trajectories of depression and anxiety in blended care therapy
Source: PLoS One. 2024 Nov 8;19(11):e0313112. doi: 10.1371/journal.pone.0313112 (PMC11548720; doi:10.1371/journal.pone.0313112)
Supplement: S1 Text — (DOCX) [file pone.0313112.s001.docx]

**Supporting Information**

**Auxiliary Analysis Incorporating Therapy Homework Completion**

There are multiple variables of interest that may impact that relationship between therapeutic alliance and clinical outcomes, but are beyond the scope of the present manuscript. Therapy homework completion is of particular interest, given that it has been consistently linked with clinical outcomes (Mausbach et al., 2010). As such, an additional analysis was conducted to examine whether homework completion was related to therapeutic alliance and clinical outcomes.

A pair of supplemental models incorporating digital exercise and digital lesson completion over the previous 7 days, as well as the prior 8-14 days as covariates at the WITHIN (Level 1) and BETWEEN (Level 2) levels were estimated. The latent variable decomposition procedure implemented by Mplus (Asparouhov & Muthén, 2018) produces Level 1 predictors whose coefficients mirror those produced by cluster mean centering, and are interpreted as pure-within person effects (i.e., change in predictor is relative to client’s own mean). This procedure automatically creates latent versions of these predictors at Level 2 that are composed solely of between-person variance. Because the Level 2 versions of these predictors are person-specific means that are informed by a client’s homework engagement throughout the entire episode of care, specifying directional regressions would violate the underlying temporal ordering of our model at Level 2 (i.e., homework assignments completed near the end of treatment cannot influence a client’s initial symptom score or their initial change in symptoms). As a result, the associations between these variables, the WAI variables, and the growth factors were specified as covariances.

***GAD-7***

For Group 1, the Level 1 covariate effects for digital exercise completion over the previous 7 days (b_Ex7-GAD_ = -0.134 [-0.254, -0.013], Z = -2.180, p = .029) and the prior 8-14 days (b_Ex814-GAD_ = -0.240 [-0.336, -0.143], Z = -4.860, p < .001) were statistically significant and indicated that completion of an exercise during the assessment interval was associated with lower anxiety. Similarly, both the previous 7 day (b_Les7-GAD_ = -0.418 [-0.530, -0.306], Z = -7.325, p < .001) and prior 8-14 day (b_Les814-GAD_ = -0.272 [-0.372, -0.172], Z = -5.338, p < .001) coefficients for digital lesson completion were significant and also indicated that greater digital lesson engagement was associated with lower symptoms. For Group 2, Level 1 covariate effects for digital exercise completion were negative in sign and significant for the previous 7 days (b_Ex7-GAD_ = -0.274 [-0.334, -0.213], Z = -8.879, p < .001) and the prior 8-14 days (b_Ex814-GAD_ = -0.242 [-0.303, -0.181], Z = -7.752, p < .001). Similarly, both the previous 7 day (b_Les7-GAD_ = -0.407 [-0.459, -0.355], Z = -15.291, p < .001) and prior 8-14 day (b_Les814-GAD_ = -0.323 [-0.380, -0.265], Z = -11.016, p < .001) coefficients for digital lesson completion were significant. The signs of these covariate effects suggest that when the client demonstrated greater homework completion relative to their own average rate of homework completion, they tended to report lower anxiety symptoms.

For Group 1, only 2 of the 8 Level 2 covariances linking homework completion to WAI change score components (i.e., *θ_T1-_*_Ex814_, *θ_Δ-_*_Les7_) were statistically significant, and the direction of these relationships suggest that higher initial working alliance is associated with slightly higher rates of exercise completion, and that greater initial increases in working alliance are associated with higher rates of lesson completion throughout the episode of care. For Group 2, 5 of the 8 Level 2 covariances emerged as significant (i.e., *θ_T1-_*_Ex7_, *θ_T1-_*_Ex814_, *θ_T1-_*_Les7_, *θ_T1-_*_Les814_, *θ_Δ-_*_Ex814_). The direction of these relationships suggest that higher initial working alliance is associated with slightly higher rates of exercise and lesson completion, and that greater initial increases in working alliance are associated with lower rates of exercise completion throughout the episode of care.

S1 Table. Key Parameters from GAD-7 Analysis

***PHQ-9***

For Group 1, the Level 1 covariate effects for digital exercise completion was not significant for the previous 7 days (b_Ex7-PHQ_ = -0.061 [-0.182, 0.060], Z = -0.992, p = .321), but was significant with a negative coefficient for the prior 8-14 days (b_Ex814-PHQ_ = -0.118 [-0.224, -0.013], Z = -2.200, p = .028). In contrast, both the previous 7 day (b_Les7-PHQ_ = -0.306 [-0.416, -0.196], Z = -5.466, p < .001) and prior 8-14 day (b_Les814-PHQ_ = -0.243 [-0.342, -0.144], Z = -4.805, p < .001) coefficients for digital lesson completion were significant. For Group 2, Level 1 covariate effects for digital exercise completion were negative in sign and significant for the previous 7 days (b_Ex7-PHQ_ = -0.181 [-0.241, -0.121], Z = -5.920, p < .001) and the prior 8-14 days (b_Ex814-PHQ_ = -0.177 [-0.240, -0.114], Z = -5.498, p < .001). Similarly, both the previous 7 day (b_Les7-PHQ_ = -0.243 [-0.295, -0.191], Z = -9.180, p < .001) and prior 8-14 day (b_Les814-PHQ_ = -0.218 [-0.277, -0.159], Z = -7.275, p < .001) coefficients for digital lesson completion were significant. The signs of these covariate effects suggest that when the client demonstrated greater homework completion relative to their own average rate of homework completion, they tended to report lower depression symptoms.

For Group 1, only 2 of the 8 Level 2 covariances linking homework completion to WAI change score components (i.e., *θ_T1-_*_Ex814_, *θ_Δ-_*_Les7_) were statistically significant, and the direction of these relationships suggest that higher initial working alliance is associated with slightly higher rates of exercise completion, and that greater initial increases in working alliance are associated with higher rates of lesson completion throughout the episode of care. For Group 2, 5 of the 8 Level 2 covariances emerged as significant (i.e., *θ_T1-_*_Ex7_, *θ_T1-_*_Ex814_, *θ_T1-_*_Les7_, *θ_T1-_*_Les814_, *θ_Δ-_*_Ex814_). The direction of these relationships suggest that higher initial working alliance is associated with slightly higher rates of exercise and lesson completion, and that greater initial increases in working alliance are associated with lower rates of exercise completion throughout the episode of care.

S2 Table. Key Parameters from PHQ-9 Analysis

Ultimately, while there were generally significant impacts of homework completion on clinical outcomes, the inclusion of these covariates had no meaningful impact on the pattern of findings described in the primary analysis.

**References for Supporting Information Section**

Asparouhov, T., & Muthén, B. (2019). Latent variable centering of predictors and mediators in multilevel and time-series models. Structural Equation Modeling: A Multidisciplinary Journal, 26, 119-142.

Mausbach, B. T., Moore, R., Roesch, S., Cardenas, V., & Patterson, T. L. (2010). The relationship between homework compliance and therapy outcomes: An updated meta-analysis. Cognitive therapy and research, 34, 429-438.
