# Supplementary material for: A large-scale evaluation of therapeutic alliance and symptom trajectories of depression and anxiety in blended care therapy
Source: PLoS One. 2024 Nov 8;19(11):e0313112. doi: 10.1371/journal.pone.0313112 (PMC11548720; doi:10.1371/journal.pone.0313112)
Supplement: S1 Table — (DOCX) [file pone.0313112.s002.docx]

S1 Table. Key Parameters from GAD-7 Analysis

|  | | | | | | | | | | | | |
| --- | --- | --- | --- | --- | --- | --- | --- | --- | --- | --- | --- | --- |
|  |  | Group 1 | | | | |  | Group 2 | | | | |
| Parm |  | Est [95% CI] |  | Z |  | p-value |  | Est [95% CI] |  | Z |  | p-value |
| b_Ex7_ |  | -0.134 [-0.254, -0.013] |  | -2.180 |  | 0.029 |  | -0.274 [-0.334, -0.213] |  | -8.879 |  | <0.001 |
| b_Ex814_ |  | -0.240 [-0.336, -0.143] |  | -4.860 |  | <0.001 |  | -0.242 [-0.303, -0.181] |  | -7.752 |  | <0.001 |
| b_Les7_ |  | -0.418 [-0.530, -0.306] |  | -7.325 |  | <0.001 |  | -0.407 [-0.459, -0.355] |  | -15.291 |  | <0.001 |
| b_Les814_ |  | -0.272 [-0.372, -0.172] |  | -5.338 |  | <0.001 |  | -0.323 [-0.380, -0.265] |  | -11.016 |  | <0.001 |
| *α_ΔWAI_* |  | 4.295 [3.961, 4.629] |  | 25.189 |  | <0.001 |  | 2.746 [2.609, 2.882] |  | 39.442 |  | <0.001 |
| *α_Int_* |  | 10.748 [10.592, 10.904] |  | 134.656 |  | <0.001 |  | 11.048 [10.963, 11.132] |  | 256.577 |  | <0.001 |
| *α_Lin_* |  | -0.983 [-1.042, -0.924] |  | -32.702 |  | <0.001 |  | -1.005 [-1.035, -0.976] |  | -67.628 |  | <0.001 |
| *α_Qua_* |  | 0.044 [0.040, 0.049] |  | 19.109 |  | <0.001 |  | 0.044 [0.042, 0.047] |  | 38.838 |  | <0.001 |
| *b_T1-Int_* |  | 0.012 [-0.005, 0.028] |  | 1.379 |  | 0.168 |  | 0.002 [-0.008, 0.012] |  | 0.347 |  | 0.729 |
| *b_Δ-Int_* |  | 0.015 [-0.009, 0.039] |  | 1.206 |  | 0.228 |  | 0.007 [-0.009, 0.023] |  | 0.833 |  | 0.405 |
| *b_T1-Lin_* |  | -0.020 [-0.026, -0.014] |  | -6.779 |  | <0.001 |  | -0.024 [-0.027, -0.021] |  | -15.187 |  | <0.001 |
| *b_Δ-Lin_* |  | -0.019 [-0.026, -0.011] |  | -4.716 |  | <0.001 |  | -0.025 [-0.030, -0.020] |  | -10.349 |  | <0.001 |
| *b_T1-Qua_* |  | 0.001 [0.001, 0.002] |  | 5.629 |  | <0.001 |  | 0.001 [0.001, 0.002] |  | 10.625 |  | <0.001 |
| *b_Δ-Qua_* |  | 0.001 [0.001, 0.002] |  | 3.892 |  | <0.001 |  | 0.001 [0.001, 0.002] |  | 7.454 |  | <0.001 |
| *θ_T1-Ex7_* |  | 0.012 [-0.077, 0.100] |  | 0.260 |  | 0.795 |  | 0.136 [0.097, 0.175] |  | 6.822 |  | <0.001 |
| *θ_T1-Ex814_* |  | 0.089 [0.000, 0.178] |  | 1.968 |  | 0.049 |  | 0.145 [0.106, 0.184] |  | 7.371 |  | <0.001 |
| *θ_T1-Les7_* |  | -0.062 [-0.155, 0.031] |  | -1.311 |  | 0.190 |  | 0.039 [0.005, 0.073] |  | 2.224 |  | 0.026 |
| *θ_T1-Les814_* |  | 0.026 [-0.056, 0.107] |  | 0.618 |  | 0.537 |  | 0.064 [0.030, 0.097] |  | 3.720 |  | <0.001 |
| *θ_Δ-Ex7_* |  | 0.027 [-0.046, 0.101] |  | 0.732 |  | 0.464 |  | -0.023 [-0.054, 0.008] |  | -1.429 |  | 0.153 |
| *θ_Δ-Ex814_* |  | -0.031 [-0.098, 0.037] |  | -0.895 |  | 0.371 |  | -0.033 [-0.062, -0.004] |  | -2.228 |  | 0.026 |
| *θ_Δ-Les7_* |  | 0.082 [0.010, 0.154] |  | 2.238 |  | 0.025 |  | 0.022 [-0.005, 0.048] |  | 1.613 |  | 0.107 |
| *θ_Δ-Les814_* |  | 0.029 [-0.032, 0.090] |  | 0.941 |  | 0.347 |  | 0.007 [-0.019, 0.033] |  | 0.543 |  | 0.587 |
