# Supplementary material for: A large-scale evaluation of therapeutic alliance and symptom trajectories of depression and anxiety in blended care therapy
Source: PLoS One. 2024 Nov 8;19(11):e0313112. doi: 10.1371/journal.pone.0313112 (PMC11548720; doi:10.1371/journal.pone.0313112)
Supplement: S2 Table — (DOCX) [file pone.0313112.s003.docx]

| S2 Table. Key Parameters from PHQ-9 Analysis | | | | | | | | | | | | |
| --- | --- | --- | --- | --- | --- | --- | --- | --- | --- | --- | --- | --- |
|  |  | Group 1 | | | | |  | Group 2 | | | | |
| Parm |  | Est [95% CI] |  | Z |  | p-value |  | Est [95% CI] |  | Z |  | p-value |
| b_Ex7_ |  | -0.061 [-0.182, 0.060] |  | -0.992 |  | 0.321 |  | -0.181 [-0.241, -0.121] |  | -5.920 |  | <0.001 |
| b_Ex814_ |  | -0.118 [-0.224, -0.013] |  | -2.200 |  | 0.028 |  | -0.177 [-0.240, -0.114] |  | -5.498 |  | <0.001 |
| b_Les7_ |  | -0.306 [-0.416, -0.196] |  | -5.466 |  | <0.001 |  | -0.243 [-0.295, -0.191] |  | -9.180 |  | <0.001 |
| b_Les814_ |  | -0.243 [-0.342, -0.144] |  | -4.805 |  | <0.001 |  | -0.218 [-0.277, -0.159] |  | -7.275 |  | <0.001 |
| *α_ΔWAI_* |  | 4.283 [3.949, 4.617] |  | 25.121 |  | <0.001 |  | 2.739 [2.603, 2.875] |  | 39.389 |  | <0.001 |
| *α_Int_* |  | 9.140 [8.898, 9.383] |  | 73.930 |  | <0.001 |  | 9.768 [9.634, 9.902] |  | 143.096 |  | <0.001 |
| *α_Lin_* |  | -0.804 [-0.864, -0.743] |  | -26.148 |  | <0.001 |  | -0.853 [-0.884, -0.823] |  | -54.471 |  | <0.001 |
| *α_Qua_* |  | 0.036 [0.032, 0.041] |  | 15.400 |  | <0.001 |  | 0.037 [0.035, 0.039] |  | 32.818 |  | <0.001 |
| *b_T1-Int_* |  | -0.021 [-0.046, 0.004] |  | -1.666 |  | 0.096 |  | -0.015 [-0.030, 0.000] |  | -1.903 |  | 0.057 |
| *b_Δ-Int_* |  | 0.017 [-0.019, 0.054] |  | 0.931 |  | 0.352 |  | 0.017 [-0.007, 0.041] |  | 1.411 |  | 0.158 |
| *b_T1-Lin_* |  | -0.013 [-0.019, -0.007] |  | -4.283 |  | <0.001 |  | -0.024 [-0.028, -0.021] |  | -12.677 |  | <0.001 |
| *b_Δ-Lin_* |  | -0.018 [-0.026, -0.010] |  | -4.217 |  | <0.001 |  | -0.026 [-0.031, -0.020] |  | -9.167 |  | <0.001 |
| *b_T1-Qua_* |  | 0.001 [0.000, 0.001] |  | 3.270 |  | 0.001 |  | 0.001 [0.001, 0.002] |  | 10.225 |  | <0.001 |
| *b_Δ-Qua_* |  | 0.001 [0.000, 0.002] |  | 3.487 |  | <0.001 |  | 0.001 [0.001, 0.002] |  | 6.945 |  | <0.001 |
| *θ_T1-Ex7_* |  | 0.012 [-0.077, 0.100] |  | 0.260 |  | 0.795 |  | 0.136 [0.097, 0.176] |  | 6.829 |  | <0.001 |
| *θ_T1-Ex814_* |  | 0.089 [0.000, 0.178] |  | 1.967 |  | 0.049 |  | 0.145 [0.107, 0.184] |  | 7.379 |  | <0.001 |
| *θ_T1-Les7_* |  | -0.062 [-0.155, 0.031] |  | -1.310 |  | 0.190 |  | 0.039 [0.005, 0.074] |  | 2.234 |  | 0.025 |
| *θ_T1-Les814_* |  | 0.026 [-0.056, 0.108] |  | 0.618 |  | 0.536 |  | 0.064 [0.030, 0.097] |  | 3.727 |  | <0.001 |
| *θ_Δ-Ex7_* |  | 0.028 [-0.045, 0.101] |  | 0.752 |  | 0.452 |  | -0.022 [-0.053, 0.009] |  | -1.370 |  | 0.171 |
| *θ_Δ-Ex814_* |  | -0.030 [-0.097, 0.037] |  | -0.873 |  | 0.382 |  | -0.032 [-0.061, -0.003] |  | -2.160 |  | 0.031 |
| *θ_Δ-Les7_* |  | 0.083 [0.012, 0.155] |  | 2.278 |  | 0.023 |  | 0.021 [-0.005, 0.047] |  | 1.583 |  | 0.113 |
| *θ_Δ-Les814_* |  | 0.031 [-0.029, 0.091] |  | 1.002 |  | 0.316 |  | 0.007 [-0.019, 0.033] |  | 0.539 |  | 0.590 |
